# In Search of Panacea—Review of Recent Studies Concerning Nature-Derived Anticancer Agents

**DOI:** 10.3390/nu11061426

**Published:** 2019-06-25

**Authors:** Dawid Przystupski, Magdalena J. Niemczura, Agata Górska, Stanisław Supplitt, Krzysztof Kotowski, Piotr Wawryka, Paulina Rozborska, Kinga Woźniak, Olga Michel, Aleksander Kiełbik, Weronika Bartosik, Jolanta Saczko, Julita Kulbacka

**Affiliations:** 1 Faculty of Medicine, Wroclaw Medical University, J. Mikulicza-Radeckiego 5, 50-345 Wroclaw, Poland; stkwiatek@gmail.com (S.S.); krzysztof.kotowski96@gmail.com (K.K.); piotr95wawryka@gmail.com (P.W.); akielbik6@gmail.com (A.K.); 2 Department of Biological Sciences, Institute of Experimental Biology, University of Wrocław, Kanonia 6/8, 50-328 Wroclaw, Poland; m.j.niemczura@gmail.com (M.J.N.); agata_gorska@onet.eu (A.G.); 3 Faculty of Chemistry, Wroclaw University of Science and Technology, Norwida 4/6, 50-373 Wroclaw, Poland; rozborska.paulina@gmail.com (P.R.); kinga.wozniak@onet.eu (K.W.); 4 Department of Medical Biochemistry, Wroclaw Medical University, Chalubinskiego 10, 50-368 13 Wroclaw, Poland; michel.olga.maria@gmail.com; 5 Faculty of Biotechnology, University of Wroclaw, Joliot-Curie 14a, 50-385 Wroclaw, Poland; weronikabartosik@gmail.com; 6 Department of Molecular and Cellular Biology, Wroclaw Medical University, Borowska 211A, 50-25 556 Wroclaw, Poland; jolanta.saczko@umed.wroc.pl

**Keywords:** natural substances, nutraceuticals, phytochemicals, chemoprevention, nutri-epigenetics, anticancer research, mitotic inhibitors, topoisomerase inhibitors, xenobiotics, 6-gingerol, honokiol, polyphenols, drug resistance

## Abstract

Cancers are one of the leading causes of deaths affecting millions of people around the world, therefore they are currently a major public health problem. The treatment of cancer is based on surgical resection, radiotherapy, chemotherapy or immunotherapy, much of which is often insufficient and cause serious, burdensome and undesirable side effects. For many years, assorted secondary metabolites derived from plants have been used as antitumor agents. Recently, researchers have discovered a large number of new natural substances which can effectively interfere with cancer cells’ metabolism. The most famous groups of these compounds are topoisomerase and mitotic inhibitors. The aim of the latest research is to characterize natural compounds found in many common foods, especially by means of their abilities to regulate cell cycle, growth and differentiation, as well as epigenetic modulation. In this paper, we focus on a review of recent discoveries regarding nature-derived anticancer agents.

## 1. Introduction

Neoplastic disease presents a considerable challenge for current health systems. Proof of this can be seen in increasing trends in annual statistics concerning incidence [1]. Currently applied strategies for cancer treatment are primarily based on surgical resection procedures of the neoplastic mass followed by the introduction of radiotherapy, immunotherapy, and chemotherapy. However, in a significant amount of cases, cancers still present only mediocre clinical response to universal protocols developed for primary tumors or metastases. Furthermore, a considerable amount of anticancer agents carry the risk of various adverse reactions, toxicity and low selectivity for tumor cells [2]. Due to numerous clinical implications arising from this phenomena, scientists from all over the world have focused on the search for novel drugs, with special emphasis on implementation of antitumor compounds from natural sources [3].

In the history of anti-cancer therapies, the majority of them have targeted the “hallmarks of cancer,” tumor-specific alterations established and described by Hanahan and Weinberg as the following: “sustaining proliferative signaling, evading growth suppressors, resisting cell death, enabling replicative immortality, inducing angiogenesis, activating invasion and metastasis, avoiding immune destruction, cancer-promoting inflammation, genome instability and mutation, and deregulating cellular energetics” [4]. Notably, recent findings regarding molecular mechanisms of carcinogenesis highlighted the importance of genetic and epigenetic alterations as an important issue in cancer prevention and treatment [5,6,7,8,9,10,11]. The considerable influence of natural compounds on epigenome has been reported [12], concerning processes associated with carcinogenesis such as histone modifications [13,14,15] (methylation, acetylation and phosphorylation) which are linked to changes in chromatin structure as well as DNA methylation [16,17] and non-coding microRNA expression [18,19,20]. Subsequently, those modifications have a broad effect on the expression of target genes, inter alia oncogenes and tumor suppression genes, hence influencing either initiation or progression of cancer.

According to the latest research, the next decade is likely to witness a considerable rise in application of nature-derived compounds exhibiting profound molecular and epigenetic activity as well in clinical cancer therapy routine [5,7,8,9,12,14,20,21,22,23,24,25,26].

The following review summarizes the anticancer activity of well-known substances of natural origin. The agents are specified below in the following order:

1. The mitotic inhibitors:
  - Vinca alkaloids–vinblastine, vincristine, vindesine, vinorelbine, vinflunine,
  - colchicine,
  - podophyllotoxin,
  - taxanes (paclitaxel, docetaxel).
2. I and II topoisomerases inhibitors:
  - camptothecin,
  - topotecan
  - irinotecan
  - etoposide
3. Inducers of xenobiotic metabolism:
  - allyl sulfide,
  - indole-3-carbinol,
  - phenethyl isothiocyanate,
  - sulforaphane,
  - glucoraphanin,
  - iberin,
  - terpenes,
  - coumarins.
4. Polyphenols:
  - carnosol
  - resveratrol
  - 6-gingerol,
  - honokiol,
  - flavonoids.

## 2. Methods

The selection of articles was conducted on the following databases: Science Direct, Scopus, PubMed, and Google Scholar. Several combinations of the following terms were used: ((“natural substances,” OR “natural compounds,” OR “plant-derived,” OR “nature-derived,” OR “phytochemicals,” OR “nutraceuticals”) AND (“cancer,” OR “tumor,” OR “metastasis”) AND (“treatment,” OR “anticancer,” “prevention,” OR “prophylaxis,” OR “cell death,” OR “drug resistance,” OR “cell cycle inhibitors”)) within the fields “article title, abstract and keywords.” Only articles published in peer-reviewed journals were chosen for the review, preferably written in English. The greatest emphasis was on articles published between 2000 and 2019, however, the specificity of this study required also referring to the earliest reports in the field. In this study, we focused on the anticancer activity of polyphenols and inducers of xenobiotic metabolism as well as natural-derived substances which have widely been used in medicine in recent years.

## 3. The Mitotic Inhibitors

Since microtubules play a significant role in mitosis and cell division, microtubule-targeted antimitotic substances have been involved in cancer therapy, since disrupting mitotic progression significantly slows down the progression of disease [27].

Anti-mitotic drugs can be divided into two major groups: microtubule-destabilizing and microtubule-stabilizing agents. The first group includes compounds such as the Vinca alkaloids (e.g., vinblastine, vincristine, vindesine or vinorelbine), colchicine and podophyllotoxin that inhibits microtubule polymerization at high concentrations. The second group consists of paclitaxel, docetaxel and epothilones which stimulate microtubule polymerization (Table 1) [28]. 

The antimitotic potential of Vinca alkaloids was discovered in the late 1950s and since then there has been a rapid rise in the use of these compounds in cancer treatment [28]. At high concentrations (e.g., 10–100 nM in HeLa cells) Vinca alkaloids depolymerize microtubules and disrupt mitotic spindles, leading to cell cycle arrest [29]. On the contrary, low but clinically significant concentrations of vinblastine (VBL) (e.g., IC50 0.8 nM in HeLa cells), do not depolymerize spindle microtubules, but extensively block mitosis leading to cell apoptosis. VBL binds rapidly and reversibly to the β-subunit of tubulin dimers at a region adjacent to the GTP-binding site, known as the *Vinca-binding domain* [27]. The binding affinity to microtubules varies among the family of Vinca alkaloids, depending on location of binding sides. Thus, at low concentrations (<1 μmol) these compounds bind to the high-affinity sites localized at the microtubule ends. At high concentrations (>1 μmol), they tend to bind to low-affinity binding sites along the microtubule surface leading to microtubule depolymerization [30,31,32]. Outstanding clinical efficacy of VBL as well as its oxidized form, Vincistrine (VCR), applied in several combination therapies, together with the desire to develop orally available analogues, have contributed to the development of various novel semi-synthetic derivatives, including vindesine (VDS), vinorelbine (VRL) and vinflunine (VFL) [33,34,35] (Table 1).

The risk of side effects and multidrug resistance has slowed down the introduction of Vinca alkaloids for clinical use. To solve these problems, researchers have developed numerous strategies, e.g., using liposomal drug delivery systems [36], chemically modified drugs, and encapsulation in polymeric nanocarriers, to reduce the toxicity and enhance the therapeutic efficiency of Vinca alkaloids [37].

Colchicine, a natural compound isolated from the poisonous meadow saffron *Colchicum autumnale* L., [33] has been approved for therapy by the U.S Food and Drug Administration (FDA) in 2009 [38] after years of successful application in the treatment of numerous diseases [39,40,41,42] (Table 1). The mechanism of action of this compound as well as final effect is quite similar to Vinca alkaloids, namely cell cycle arrest which is caused by depolymerization of the microtubules at high concentrations and stabilizing their dynamics at low concentrations [43]. Colchicine also has the potential to affect intracellular tubulin which leads to restricting mitochondrial metabolism in cancer cells by inhibiting the voltage-dependent anion channels that are located in the mitochondrial membrane [44]. Relatively low therapeutic index did not allow the implementation of colchicine in routine cancer treatment [45]. However, studies using nanoparticle-mediated targeted delivery of colchicine shed a new light on this case, allowing the toxic effects of colchicine to be circumvented [46]. Tangutoori et al. (2014) have used Pegylated Cationic Liposomal-colchicine (PCL-colchicine) nanoparticles for in vitro and in vivo studies of lung cancer, and have demonstrated that microtubules are more effectively disrupted by nanoparticle-loaded colchicine than colchicine in natural form. An in vivo study has shown that accumulation of PCL-colchicine in the malignant lung enhanced twofold in comparison to the normal lung, providing longer survival time for the group treated with the PCL-colchicine [47].

Podophyllotoxin (PPT), an aryltetralin lignan naturally occurring in *Podophyllum peltatum* and *L., Podophyllum emodi*, that effectively binds to the colchicine-binding side of tubulin from the mitotic spindle, precluding polymerization into microtubules [48]. Due to severely toxic side effects, PPT is unsuitable for clinical use as an antitumor agent [49]. However, in recent decades, a few semi-synthetic derivatives of podophyllotoxin ex etoposide and tenoposide have proven effective in cancer therapy [50]. The mechanism of action of these compounds is based on stabilization of the covalent bond between DNA and topoisomerase II projecting on inhibition of this enzyme, thus preventing the re-ligation of DNA [51]. 

## 4. I and II Topoisomerases Inhibitors 

Topoisomerases are enzymes found in the nucleus responsible for reducing torsion in supercoiled DNA. This activity is essential for intracellular processes such as replication, recombination, repair and transcription [76]. Their mechanism of action is based on the repetitive disruption and following ligation of DNA strands [77]. There are two main classes of topoisomerases: type I and type II, both potential molecular targets for anticancer therapeutics. Topoisomerase I relaxes the tension and torsion of the dsDNA using the “controlled rotation” mechanism rather than strand passage by inducing single strand, reversible breaks. Type II topoisomerase family, on the other hand, untangles and relaxes the DNA molecule by passing one strand through the opening they have cleaved beforehand and then resealing the break [76]. 

Camptothecin is quinoline alkaloid derived from *Camptotheca acuminata* known for its inhibitory effect on topoisomerase I [78]. Due to severe side effects, preliminary testing of camptothecin-based therapies were eventually abandoned until the the late eighties [78], having been replaced by administering of semisynthetic derivatives like topotecan [79]. This progress allowed the extension of treatment regimens to other agents effective against colorectal cancer [80], small cell lung cancer [81] and leukemia (Table 2).

Among topoisomerase II inhibitors, etoposide (podophyllotoxin ligand derivative), present in *Podophyllum peltatum,* is worth particular attention due to its widely described antineoplasic properties (Table 2). Dimeric tetrahydroxanthones, isolated from fungus *Aspergillus versicolor,* are worth mentioning as a new class of potent topoisomerase I inhibitors. They inhibit topoisomerase I-mediated DNA relaxation, induce cell cycle arrest and necrosis of cancer cells [82].

## 5. Inducers of Xenobiotics Metabolism

The enzymes in the metabolic pathway of xenobiotics play a significant role in the prevention of carcinogenesis. It allows for the detoxification and elimination of potentially dangerous chemicals from the body. The oncogenic effect of xenobiotics is neutralized by their biotransformation with the participation of oxidoreductases and transferases in a process consisting of two phases: phase I and phase II [2]. Phase II enzymes are responsible for the so-called detoxification phase, during which xenobiotics are transformed into their variant forms, facilitating their excretion from the body [89]. This process involves the coupling of xenobiotics with endogenous metabolites such as glutathione, glycine as well as glucuronic, acetic, glutamic and sulfuric acids. The coupling reactions lead to the increased solubility of xenobiotics in body fluids, allowing them to be excreted via urine [90].

Glutathione S-transferases (GSTs) are among the most crucial enzymes for the second phase of xenobiotics biotransformation. They catalyze the coupling reactions between reduced glutathione and electrophilic intermediate metabolites formed as a result of the phase I reactions [91]. The coupling of xenobiotics with glutathione, in most cases, leads to their inactivation and reduces their cytotoxic activity [92]. The chemopreventive properties of GSTs play an important role in the regulation of carcinogenesis. Another very important characteristic of GSTs is their participation in cellular defense against reactive oxygen species (ROS), which damage various macromolecules present in the cell, such as nucleic acids, proteins, lipids, and are responsible for the development of many pathological conditions—including cancers [93].

**Figure 1:**
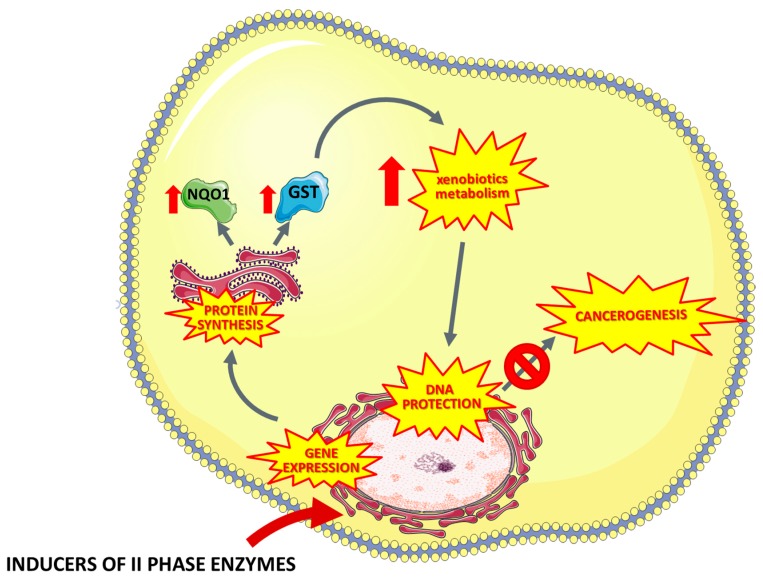
Mechanisms of the chemopreventive activity of the phase II detoxification enzymes (such as organosulfur compounds indole-3-carbinol, isothiocyanates, sulforaphan, glucoraphanin, iberin, phenolic compounds, terpenes, coumarins) leading to inhibition of carcinogenesis. NQO1— NAD(P)H dehydrogenase (quinone 1), GST—Glutathione S-transferase.

Another important enzyme involved in the detoxification of xenobiotics is NADPH: quinone oxidase (NQO1). The main function of NQO1 is the reduction of endogenous and exogenous quinones as well as quinone compounds to hydroquinones [94,95]. Many studies have shown that selective induction of phase II enzymes leads to the cell protection against xenobiotics and reactive oxygen species [96,97,98,99]. Thus, modulation of the expression of phase II enzymes may be an important element of the chemopreventive strategy (Figure 1, Table 3).

### 5.1. Terpenes

Terpenes (isoprenoids) are a diverse and highly varied family of chemical compounds widely distributed in nature, possessing a broad range of pharmacological and biological properties [100]. They are an example of compounds that induce the synthesis of phase II enzymes by activating the Nrf2 protein (nuclear erythroid 2-related factor) [101]. Nrf2 is a transcription factor that activates many genes encoding proteins such as GST, NQO1 [102]. Studies confirm that diterpenes, kahweol and cafestol, isolated from green coffee seeds, are capable of inducing GST activity in various tissues in mice and increase levels of GST in rat liver and kidneys [103,104].

#### 5.1.1. Parthenolide

A sesquiterpene lactone isolated from Tanacetum parthenium—a parthenolide—can be distinguished by its broad anti-cancer properties on the cellular as well as epigenetic level [11,13]. Numerous studies reported its involvement in inhibiting NF-kB activation, promotion of cell differentiation, cell cycle arrest or induction of apoptosis [105,106,107,108,169]. Apart from these actions, it has been shown to specifically deplete HDAC1 protein (Histone deacetylase 1), causing p53 activation through ubiquitination of E3 ubiquitin-protein ligase MDM2, overall reflecting on DNA damage response [109,110]. Parthenolide has been reported to inhibit DNMT1 (DNA (cytosine-5)-methyltransferase 1) activity as well as decrease DNMT1 expression which is linked to the decrease the global DNA methylation, leading to hypomethylation and activation of the HIN-1 tumor suppressor gene in cellular model of leukemia and breast cancer [111]. 

#### 5.1.2. Betulinic Acid

Betulinic acid (BA) is a pentacyclic triterpene derived from the bark of *Betula pubescens*, *Betula pendula*, *Betula humilis* and *Betula nana[34x]* but also from a variety of tropical plants such as *Syzygium formosanum*, *Tryphyllum peltatum*, *Diospyros leucomelas*, *Tetracera boliviana*, *Ancistrocladus heyneaus* and *Zizyphus joazeiro* [170]. Betulinic acid was proven to induce apoptosis via the mitochondrial pathway by upregulation of the concentration of intracellular ROS level [112]. Interestingly, in cells preincubated with an antioxidative solution, the reaction was not observed [113].

Moreover, betulinic acid has been reported to have no negative effect on normal cells, which is a quality often desirable in anticancer treatment. Its selective cytotoxicity has been tested on different human tumor cell lines in comparison to doxorubicin, a cytostatic agent commonly used in anticancer treatment. Betulinic acid exhibited up to 2–5 times lower cytotoxicity than doxorubicin with doses IC_50_ 10 μg/mL and IC_50_ 0.38 μg/mL, respectively on human normal derma fibroblasts. When tested on peripheral blood lymphocytes (PBL), the difference between betulinic acid and a commonly used anticancer agent was even more significant, showing up to 1000 times less cytotoxicity with tested dosages of doxorubicin and betulinic acid being IC_50_ 50 μg/mL, IC_50_ 0.058 +/− 0.008 μg/mL, respectively [170].

Betulin itself is inactive when applied to specific cancer cell lines, such as melanoma, neuroblastoma, leukaemia or epidermoid carcinoma. However, it can be easily converted to betulinic acid which exhibits anticancer properties. According to this research, the cytotoxic properties of betulinic acid increase with the decrease in intracellular pH [114].

**Figure 2:**
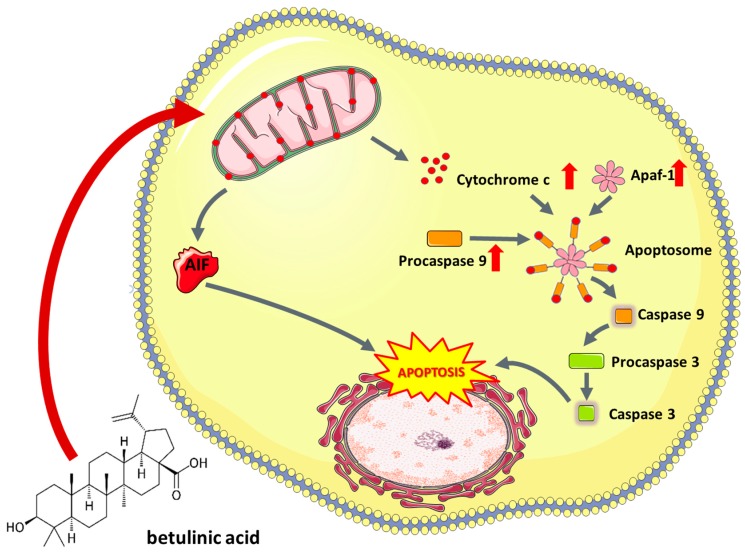
Proapoptotic activity of betulinic acid observed in cancer cells. AIF—Apoptosis Inducing Factor, Apaf-1—Apoptotic protease activating factor 1.

The broad spectrum of betulinic acid’s mechanisms of action is still being studied, although there is an implication of propensity to interfere with mitochondrial stability by increasing permeabilization of its membrane, which leads to induction of mitochondrial apoptosis pathway [112]. Permeabilization of mitochondrial membrane results in the release of cytochrome c or apoptosis inducing factor (AIF) to the cytoplasm, where they have ability to activate caspase cascade inevitably triggered to nuclear fragmentation. Antiapoptotic factors, such as Bcl-2 or Bcl-X_L,_ suppress apoptosis by stabilizing the mitochondrial membrane, thereby preventing it from permeabilization [115] (Figure 2). Betulinic acid’s way of induction of apoptosis is different from those induced by doxorubicin and other anticancer agents, since it does not affect ligand/receptor systems such as CD95 or p53 protein [115], but rather interfere with the continuity of mitochondrial membrane which results in the release of cytochrome c [116].

Combination of betulinic acid and other anticancer agents, like vincristine, may give a wide range of different cytotoxic effects [114]. Both are used to induce cell cycle arrest in murine melanoma cell line B16F10, at G1 phase and G2/M phase respectively, therefore one augments the effect of the other [117] which results in induction of programmed cell death. Combination of betulinic acid and tumor necrosis factor (TNF)-related apoptosis-inducing ligand (TRAIL) applied to human neuroblastoma cell line (SHEP), leads to apoptosis of the cells [114,171]. Interestingly, in normal cells that effect did not take place. Betulinic acid, doxorubicin, VP16, taxol and actinomycin D induced apoptosis in the SHEP cell line [114]. In some cases, betulinic acid can work as a cell sensitizer for other anticancer agents, such as doxorubicin, in a wide range of cell lines including human melanoma cells MelJuSo, glioblastoma A172 or medulloblastoma Daoy [114].

Recent emergence of a new drug delivery system using gold-based nanomaterials gives a new hope for precise drug dosage due to its low toxicity and photothermal responsive properties, different from properties of the previous gold nanoparticle systems. Different shapes of gold particles have been tested in terms of the photothermal response. Gold nanoshells have been shown to present the most optimal properties for therapy [172]. The new drug delivery system consists of liposomes containing betulinic acid coated with gold particles. BA (betulinic acid) is known from its highly lipophilic properties, which results in its limited bioavailability [173]. The new method of encapsulation of the acid inside liposomes seems to increase the solubility of the particle.

Another way of BA delivery to the tumor tissue may be carbon nanotubes, already widely used in medicine for direct drug delivery. Binding of the BA to nanotubes, called MWCNT-BA, can increase its absorption into the target tissue [174]. The -OH group of BA forms a non-covalent bond with the –COOH group on the external surface of a nanotube. Due to the non-covalent nature of the bond between BA and a nanotube, the release of BA through desorption is facilitated.

### 5.2. Coumarins

Coumarins are glycosides widely present in plant tissues. They are found in citrus fruits and some vegetables such as parsley, celery and parsnips [118]. Van Lieshout et al. (1998) showed that coumarins administered through diet (at a dose of 2500 mg/kg) increase the level of glutathione S-transferase in the esophagus, stomach and intestine rat test subjects. [137]. Moreover, auraptene (coumarin present in orange peel), apart from showing protective properties in the tumorigenesis induced by 7,12-dimethylbenzanthracene in the skin of mice [119], significantly increased GST and NQO1 activity in the liver and colon of rats after oral administration [120].

Some initial studies on dihydrocoumarin (DHC) derived from *Melilotus officinalis* (sweet clover) indicated that coumarins may be involved in epigenetic regulation of proteins involved in carcinogenesis. It has been reported that DHC inhibits SIRT1 deacetylase, leading to concentration- dependent increase in p53 acetylation which contributed to the cytotoxic effect in human lymphoblastoid cell line TK6 [175]. More recent studies conducted on yeast suggested that DHC possesses HDAC inhibitor activity which is associated with inhibition of Rad52, critical to double-strand repair and DNA damage sensitivity [176]. It has been demonstrated that coumarin-based analogues are endowed with HDAC inhibitory and antitumor properties [177]. In addition, recently derived by Abdizadeh et al., coumarin-based benzamides have exhibited significant cytotoxicity and potent HDAC inhibiting activity against six human cancer cell lines (HCT116, A2780, MCF7, PC3, HL60 and A549) [178].

### 5.3. Isothiocyanates

Organic isothiocyanates, commonly found in human diet, are responsible for the spicy, burning taste and aroma of certain foods, such as mustard, wasabi and horseradish [179]. In general, isothiocyanates are breakdown products of glucosinolates induced by plants as a defensive response. The process is often catalyzed by the enzyme myrosinase. Isothiocyanates exhibit strong upregulation of GST and NQO1 in murine models [180].

#### 5.3.1. Phenethyl Isothiocyanate

Among isothiocyanates, phenethyl isothiocyanate (PEITC) is of the greatest medical interest. It has been proven to have an anticancerogenic effect in an N-methyl nitrosourea-induced breast cancer animal model [121]. It is worth noting that particularly strong activity of PEITC was observed in melanoma models [122,123]. Numerous studies indicate that PEITC has also chemopreventive properties in vitro against: aforementioned breast cancer [124,125], cervical cancer [126], osteogenic sarcoma [127], prostate cancer [128] and myeloma cell lines [129]. Furthermore, PEITC promotes apoptosis by activating caspase-dependent pathways [130]. Therefore, we can conclude that it is not only a chemopreventive substance, but also an active cytotoxic agent that can be utilized against cancer cells.

Wang et al. were among the first to describe the epigenetic modifications caused by PEITC in prostate cancer LNCaP cells, discovering that PEITC reactivates the expression of glutathione S-transferase gene (GSTP1) through demethylation of the GSTP1 gene promoter [131]. The same study demonstrated that PETC influenced histone acetylation and methylation patterns as well as inhibited the activity of HDACs. Recent investigations revealed another epigenetic pathway influenced by PEITC in LNCaP cells, targeting in RASSF1A promoter methylation by DNA methyltransferases (DNMT1, 3A and 3B), resulting in CpG demethylation of those regions, while inhibition of HDAC1, 2, 4 and 6 protein expression was also confirmed [132]. On the other hand, an early study on DS19 mouse erythroleukemia cells treated with allyl isothiocyanate has shown an increase in acetylation of histones without any significant effect on HDACs [133]. In another studies, the modulatory potential of pre-treatment with PEITC on expression of miRNA induced by cigarette smoke was investigated on rats in vivo. A broad spectrum miRNAs affected by PEITC was detected, namely: miR-125b miR-26a, miR-146-pre, let-7a, let-7c, miR-192, miR-222-pre, miR-99 and miR- 123 linked to the TGF-β expression, NF-κB and Ras activation, as well as cell proliferation, apoptosis and angiogenesis [134]. A similar trial with cigarette smoke was performed on mice to investigate the effect of PEITC along with glucocorticoid budesonide in different combinations. The effect of PEITC on miRNA expression differed among the organs—significant downregulation of nine and upregulation of three miRNAs was observed in the liver while miRNA expression in the lungs was rather moderate. Affected miRNAs were involved in the regulation of stress response, protein repair, cell proliferation, and inflammation [135]. More recent evidence indicates that PEITC may suppress prostate cancer cell invasiveness epigenetically through microRNA-194 mediated downregulation of BMP1, thus resulting in decreased expression of MMP2 and MMP9 (key oncogenic matrix metalloproteinases) [136].

Isothiocyanates are used in the production of nanomolecules. Fluorescein isothiocyanate (FITC) has also found application in biomedical imaging techniques [181]. Studies on the use of FITC with other particles for diagnostic and therapeutic purposes in oncology are currently underway [182,183].

#### 5.3.2. Sulforaphane and Glucoraphanin

Sulforaphane (SFN), an isothiocyanate isolated from broccoli, is one of the strongest natural inducers of GSTs and NQO1 [138]. It can be found at high concentrations in mature plants, and in broccoli sprouts up to several days after germinating [139]. The chemopreventive properties of sulforaphane have been confirmed on both in vitro and in vivo animal models [140]. The oncoprotective action of sulforaphane is based on the induction of the glutathione S-transferase in liver cells [137,141]. Many in vitro studies confirm the effective, proapoptotic action of sulforaphane.

The plant precursor for sulforaphane is glucoraphanin, which has weaker chemopreventive properties [142]. Sulforaphane is a product of an enzymatic hydrolysis of glucoraphanin performed by myrosinase, an enzyme released during disintegration of a plant tissue. Unfortunately, during cooking it undergoes denaturation, which reduces the efficiency of active sulforaphane formation. However, due to the enzymatic activity of the microflora living in the large intestine, it is possible to convert glucoraphanin into active isothiocyanates in the final section of the human gastrointestinal tract [143]. Nevertheless, Shapiro et al. (2006) showed that sulforaphane formed, with the participation of intestinal bacteria, is characterized by a six-fold decrease in bioavailability than the compound produced with the participation of the enzyme myrosinase [144]. Therefore, it is beneficial to consume broccoli in a raw or steamed form.

Comprehensive analysis of the transcriptome of Caco-2 cell line treated with SFN has revealed its complex effect on numerous genes linked to carcinogenesis, inter alia transcription factor 2 (*CDX-2*), *KLF4*, *KLF5*, cyclin-dependent kinase inhibitor 1A (*p21*), and *AMACR*, as well as downregulation of the *DNMT1* gene [184]. Another in vitro study on breast cancer cells has addressed epigenetically regulated inhibition of hTERT (human telomerase reverse transcriptase) and downregulation of DNMTs (1 and 3a) along with numerous modifications in acetylation and methylation of histone chromatin in hTERT promoter region [185]. It has been reported that due to its inhibitory effect on HDAC, SFN treatment increases the intracellular level of acetylated histones bound to p21/waf1 promoter. Interestingly, compared to SFN, HDAC inhibition was observed to be more effective in SFN metabolites, SFN-cysteine and SFN-N-acetylcysteine [186]. In other studies HDAC inhibition was linked to induction of p21 and Bax expression leading to cell cycle arrest and apoptosis [187]. Notably, a study on breast cancer confirming the inhibition of HDAC activity by SFN was conducted, but no changes in H3 or H4 acetylation were observed after exposure to the compound [188]. In a genome-wide in vitro study on prostate epithelial normal and cancer cells, Wong et al. has proven that SFN affects methylation patterns among promoter regions of cancer-associated genes [189]. In a seminal in vivo study on mice, the inhibitory role of SFN treatment on tumor transformation was addressed with a special emphasis on epigenetic mechanisms associated with anticancer genes such as Nrf2. A stimulating effect was reflected in enhanced nuclear translocation of Nrf2 as well as increased mRNA and protein levels of the Nrf2 target genes (HO-1, NQO1, UGT1A1). Epigenetic background of these changes was confirmed by a decrease in the methylation ratio of the Nrf2 gene promoter compared to control, along with decreased histone deacetylase (HDAC) activity and expression of HDACS (1,2,3,4) as well as reduced expression of DNMTs (1, 3a and 3b) [190]. Recent in vivo studies have provided another strong indication suggesting the involvement of SFN [191] and its metabolites [192] in epigenetic pathways such as inducing acetylation of histones and inhibiting HDAC activity. Recently, SFN was found to be able to restore the miR-9-3 level in A549 cells in vitro through epigenetic regulation of CpG methylation, hence providing another basis for previously described mechanisms involving DNMT and HDAC activity [193]. Interestingly, an experiment on a murine model [194] as well as in a human trial, reported a decreased HDAC activity in PBMCs after consumption of broccoli sprouts [195].

#### 5.3.3. Iberin

Iberin is a natural isothiocyanate found in horseradish [196]. A study conducted by Jakubikova et al. (2006) established that it is a potent inducer of GSTs and NQO1 in vitro. In addition, the antineoplastic effect of iberin is intensified by its action on post-translational histone modification and the induction of apoptotic cell defects [197]. Furthermore, in vivo studies showed that iberin administration to rats resulted in increased expression of detoxifying phase II enzymes (GST and NQO1) [198].

### 5.4. Organosulfur Compounds

Organic sulfur compounds and plant phenolic compounds are usually found in everyday diet [145]. Activity of enzymes in the second phase of biotransformation of xenobiotics is increased by the action of sulfur compounds present in vegetables of the genus *Allium*, e.g., garlic and onion [146]. Of the natural ingredients in *Allium* vegetable extracts, allyl sulfide, diallyl disulphide and diallyl trisulphide were proven to significantly increase GST and NQO1 activity in rat liver and colon cancer [147,148].

A growing body of literature has shown that increased histone (H3/H4) acetylation can be triggered by OSCs through targeting HDACs [149,150,151,152,153]. An in vitro study on colon cancer has presented DADS as a factor inhibiting cell proliferation and causing cell cycle arrest by triggering a decrease in HDAC activity linked to the histone hyperacetylation accompanied by p21 (Waf1/cip1) expression [153]. DADS metabolite, allyl mercaptan (AM), was shown to be a main contributor to the increase in acetylation of histones in cellular chromatin, reflecting in an accelerated binding of a SP3 transcription factor, followed by recruitment of p53 at the p21/waf1 promoter [151]. Furthermore, compared to several other OSCs, AM has demonstrated the strongest inhibitory potential on HDAC, which was confirmed in subsequent in silico studies.

### 5.5. Indole-3-Carbinol

Indole-3-carbinol (I3C) is present in a form of glucosinolate in vegetables from the *Cruciferae* family, such as cabbage, kale, brussels sprouts, broccoli [199]. Diindolylmethane (DIM) is a derivative of I3C, formed as a result of its condensation in the acidic environment of the stomach [154]. Both compounds are currently under examination due to their chemo- and oncopreventive properties. Administration of I3C upregulates the activity of NQO1 and GST in rat liver [155]. Both DIM and I3C induce the activity of the second phase enzymes (GST, UGP), showing a synergistic effect with isothiocyanates [156]. It has been proven that DIM and I3C show higher activity in chemoprevention in hormone-dependent tumors such as breast, prostate or ovarian cancer [157]. A vast number of studies have been carried out that confirm the antineoplastic properties of both I3C and DIM. In addition to chemopreventive properties, I3C inhibits the hormonal response in prostate cancer (androgenic response) and in cervical and breast cancer (estrogenic response) [158,159,160,200].

Some experiments performed on in vitro and in vivo cancer models have reported that DIM is involved in selective proteasomal degradation of class I HDACs (HDAC-1, -2, -3 and -8), while II class HDACs remained unaffected [161]. That phenomenon was associated with abolition of transcription repression of Cdks inhibitors p21/waf1 and p27/Kip2, resulting in cell cycle arrest and DNA damage triggered apoptosis. Beaver et al. evaluated the effect of I3C and DIM on prostate cancer in vitro (LNCaP, PC-3 cells, differing in the androgen receptor expression profile), observing the inhibitory effect of DIM on HDAC in both cell lines [162]. On the other hand, I3C has a moderate effect on LNCaP cells, while the PC-3 cells are indifferent to the compound. Another study has investigated the differences in miRNA expression profile in gemcitabine-sensitive and gemcitabine-resistant pancreatic cancer cells treated with DIM, confirming the upregulation of the members of miR-200 and let-7 families, which reflects on downregulation of numerous cancer-related genes [163]. Several other studies have confirmed the effect of DIM on miRNA-mediated downregulation of several cancer-related cell pathways [164,165] including genes linked to the cell invasion: EGFR, MTA-2, IRAK-1, and NF-κB [164]. I3C has been proven to downregulate miR-21 along with affecting PTEN/AKT signaling pathway in vivo [166].

## 6. Polyphenols

Polyphenols are organic compounds containing at least one aromatic ring with one or more hydroxyl functional groups attached. Polyphenols are divided into six groups: flavonols, flavones, isoflavones, flavanones, anthocyanidins, and flavanols (catechins and proanthocyanidins). They belong to a large group of plant secondary metabolites ranging from small molecules to highly polymerized compounds. These substances display many anticarcinogenic properties including inhibitory effects on proliferation of cancer cells, tumor growth, angiogenesis, metastasis, inflammation and induction of apoptosis. Moreover, numerous studies have demonstrated that natural polyphenols could be used for the prevention and treatment of cancer. Additionally, they modulate the immune system response and protect normal cells against damage caused by free radicals. There are many polyphenols which demonstrate anticancer properties, e.g., phenolic acids and their analogues (curcumin, capsaicin, 6-gingerol), tannins (trans-resveratrol), flavonoids (catechins, naringenin, theaflavin), sesamol, coumarin, tannic acid, carnosol etc. In this Section, we focus on the specific properties of 6-gingerol, honokiol and flavonoids to demonstrate the diverse biological activity of polyphenols (Table 4).

### 6.1. Carnosol

Carnosol, a natural compound found in rosemary (*Rosmarinus officinalis*), oregano (*Origanum vulgare*), and sage (*Salvia carnosa*) [201,202], has been proven to have antioxidant, anti-inflammatory and anticancer properties in animal models. Due to its strong inhibitory effect on the TPA-induced activation of epidermal ornithine carboxylase activity, carnosol is able to inhibit the development of a variety of papillomas when applied to the skin in murine models [203,204]. Carnosol is capable of halting multiple intracellular pathways, such as the JAK2-STAT3 pathway, which results in inhibition of proliferation of cancer cells by suppression of expression of cyclins and cyclin dependent kinases, and MAPK, Akt pathway, which leads to inhibition of inflammatory response via suppression of NF-κβ, COX-2 or AP-1 [203]. On the other hand carnosol, is capable of stimulating the activity of the p53 protein which leads to induction of apoptosis via activation of proapoptotic proteins [203]. When it comes to brain tumors, carnosol has been shown to be able to sensitize the glioblastoma multiforme (GBM) cells to chemotherapy by activating the p53 dependent apoptotic pathway [205]. Glioblastoma multiforme is one of the most aggressive and invasive cancers. The main aim of the treatment is to target the CSC cells, a subpopulation of cancer cells having proliferative, multipotent and self-renewal properties. Research has shown that the classic antitumor agent used to treat gliomas has a better effect on cancer cells when coupled with carnosol [206]. Furthermore, carnosol reduces CSC’s self-renewal ability.

Carnosol has antiproliferative effects on breast cancer cells expressing the estrogen receptor (ER) [205]. In the JB6 cell line, carnosol was shown to halt the Ras/ERK pathway by inhibition of RSK by directly binding to it. In gastric cell lines, carnosol has been proven to accumulate cells in the G2 phase of the cell cycle, causing cell cycle arrest. As a result, expression of cyclin B1 and protein p53 was increased [207].

Interestingly, there is a difference in the performance of carnosol as an anticancer agent between intraperitoneal delivery of the compound and dietary delivery. The former showed significant anticancer properties, whilst the latter failed to exhibit such properties [208] which may suggest differences in bioavailability of carnosol after dietary administration. However further investigation of the subject revealed that a two-week dietary administration of rosemary extract can impede the development of a mammary gland tumor in rodent models. Both carnosol and carnosoic acid induced apoptosis in B-lineage leukemia cells in vitro by downregulating the Bcl2 protein, which results in cell cycle arrest in the phase G2/M [209]

The most efficient method of administering carnosol is still to be determined. Among the tested ways of administration, DMSO was proven to be the least cytotoxic solvent for carnosol. When dispersed in liposomes, carnosol had an antiproliferative, but also cytotoxic effect on the peritoneal macrophages, where proliferation was reduced to 60% compared to control. However, this effect was only noticeable at carnosol concentration of 2,79 mg/mL, while at 0,04 and 0,17 mg/mL the effect was not observed. Carnosol dissolved in DMSO showed no cytotoxic effect, but also the proliferation of the cells was not affected [210]. The synergistic effect of carnosol and other plant derived compounds has been evaluated and phytochemicals such as capsaicin, quercetin and rosmarinic acid, show a limited anticancer activity on their own, but exhibit a stronger effect when combined with carnosol [209].

### 6.2. Resveratrol

Resveratrol (3,5,4′-trihydroxystilbene) is a phenolic compound existing in cis- and trans- isomeric forms, that is synthesized by plants in an event of fungal attack or injury. It has been used in traditional oriental medicine, extracted from Polygonum cuspidatum root [211]. However, it is also abundant in the skin of *Vitis vinifera* (common grape). Moreover, it is present in lower quantities in dietary products, such as cranberries, bilberries, blueberries, peanuts and pistachios [212,213,214]. Resveratrol drew scientists’ attention after epidemiological studies revealing cardioprotective properties of red wine [215]. In addition, clinical trials show its neuroprotective and antidiabetic properties, a positive role in non-alcoholic fatty liver diseases treatment, and an ability to act against different cancer types (prostate, breast, colorectal) [216]. A molecular mechanism underlying the anticancer properties of resveratrol is the targeting of COX proteins to downregulate tumor proliferation by inhibition of the inflammation process. Additionally, it downregulates transcription factors such as NF-κB and AP-1. Resveratrol induces cell cycle arrest and leads to apoptosis via the upregulation of survivin and Bcl2, while downregulating both BAX and p53. Resveratrol is also capable of targeting hormone signaling and due to its anti-estrogenic properties, finds a use in the treatment of hormone-dependent cancers. Chemopreventive properties are obtained by downregulation of HIF-1α as well as MMPs which influences angiogenesis and metastasis of a tumor [217]. However resveratrol might be an overall health booster by activating the MPK/SIRT1/PGC-1α pathway and, similarly to serum starvation, downstream activating key stress signaling pathways connected to TyrRS–PARP1–NAD1, which promotes metabolic health and longevity [218,219].The long-term knowledge about activation of sirtuin 1—a deacetylase—makes resveratrol one of the earliest nutraceuticals associated with epigenetic activity. It was described in literature as de-repression factor of tumor suppressors such as BRCA-1, NRF2 and RASSF-1α by methylation, PAX1 by acetylation and PTEN by both methylation and acetylation, in addition to the epigenetic regulation of oncogenic NF-κB and STAT3 signaling [220]. Furthermore, resveratrol has a positive influence on alteration of the miRNA expression ratio—high level of oncogenic miRNAs and low expression of tumor-suppressive miRNAs are commonly observed in cancer cells, thus significantly contributing to inhibition of tumor development and progression [221,222]. Resveratrol has also been demonstrated as weak DNMTs inhibitor [223]. Vergara et al. performed a proteome analysis on OVCAR-3 ovarian cancer cells treated with the compound, proving its ability to downregulate post-transcriptional cyclin D level. After showing its influence on Akt/GSK and ERK signaling pathways responsible for cyclin D1 phosphorylation and degradation, they suggested the poliphenol’s future clinical use in association with other drugs targeting Akt/GSK and ERK [209]. Although absorption of resveratrol administered orally reaches up to 75%, the bioavailability is lesser than 1% because of intensive metabolism in the intestine and liver. To avoid this disadvantageous process, a vast number of nanoformulations were produced and tested. They include liposomes, polymeric nanoparticles, solid lipid nanoparticles and cyclodextrins. None of the carriers are perfect so far. For instance, the main drawback of liposomes and solid lipid nanoparticles is low loading capacity, whereas cyclodextrins exhibit poor cancer targeting. Nevertheless, continued research in nanomaterial synthesis field may provide an attractive delivery system [224].

### 6.3. 6-Gingerol

Ginger (*Zingiber officinale* Roscoe) is a tropical plant originally found in Southeast Asia [225]. The aromatic rhizomes of ginger, both fresh and processed, are commonly used not only as a spice or a dietary supplement, but also in medicine [226]. In particular, ginger has been used in traditional oriental medicine to cure various symptoms such as rheumatic disorders and muscle pain [227]. Many beneficial properties were attributed to this plant including anti-inflammatory, antioxidative, anticancer, antimicrobial, antifungal and antiviral activity [228].

**Figure 3:**
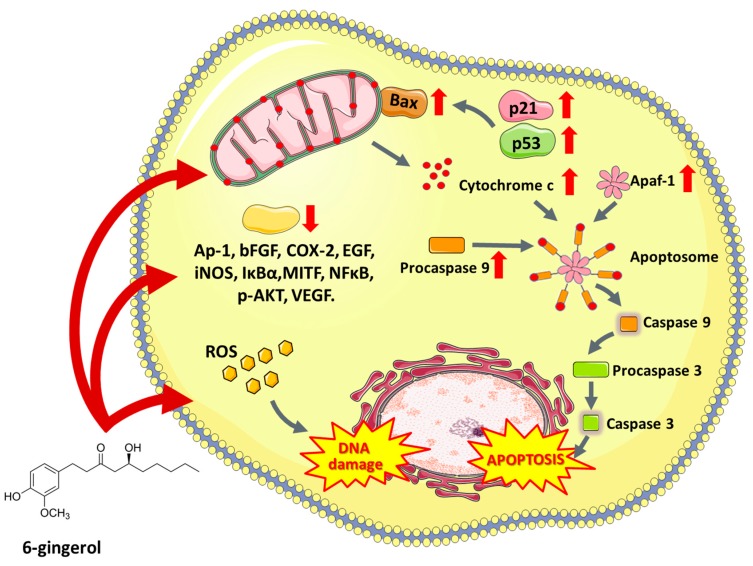
The multi-level effect of 6-gingerol (5-hydroxy-1-(4′-hydroxy3′-methoxyphenyl)-3-decanone) on cancer cells. Apaf-1—Apoptotic protease activating factor, Ap-1—Activator protein 1, Apaf-1—Apoptotic protease activating factor 1, Bax—BCL2 associated X protein, bFGF—basic fibroblast growth factor, COX-2—Prostaglandin-endoperoxide synthase 2, EGF—Epidermal growth factor, iNOS—Nitric oxide synthases, IκBα—Nuclear factor of kappa light polypeptide gene enhancer in B-cells inhibitor, alpha, MITF—Microphthalmia-associated transcription factor, NFκB—Nuclear factor kappa-light-chain-enhancer of activated B cells, p-AKT—Protein kinase B (phosphorylated), VEGF—Vascular endothelial growth factor.

6-Gingerol (5-hydroxy-1-(4′-hydroxy3′-methoxyphenyl)-3-decanone) is one of the most abundant constituents of fresh ginger and is responsible for most therapeutic properties of this plant [229]. Numerous studies have shown that 6-gingerol effectively inhibits COX-2 induction [230], hyperproliferation and inflammatory processes [231]. Moreover, this compound inhibits angiogenesis [232] and metastasis [233]. Due to antitumor and proapoptotic potential, 6-gingerol has been tested in vitro in a wide variety of cell lines, e.g., leukemia [234], breast [235], endometrial prostate [236], liver [237], colon [238], glioblastoma [239] and pancreatic cancer cell lines [240]. 6-Gingerol affects numerous pathways associated with cell death, oxidative stress, cell division and growth processes. It was reported that 6-gingerol works through the inhibition of inducible nitric oxide synthase (iNOS) [234], suppression of I-κBα [241], nuclear activation of NFκB, translocation of protein kinase C (PKC-α) [242], caspase activation [243], release of cytochrome c, increase in the expression of apoptotic protease-activating factor-1 (Apaf-1) [244], induction of oxidative stress, DNA damage [234], autophagy induction and activation of tumor suppressor proteins including p53 and p21, which leads to apoptosis (Figure 3). The ability of 6-gingerol to suppress angiogenesis is associated with inhibiting both the VEGF- and bFGF-induced proliferation of human endothelial cells and cell cycle arrest in the G1 phase [232]. Research conducted by Bode et al. showed that 6-gingerol could also block EGF-induced cell transformation and inhibit of AP-1 activation [245].

The data describing the activity of 6-gingerol as a epigenetic modulator is scarce, however Rastogi et al. suggested that apoptosis of myeloid leukemia cells treated with 6-gingerol was associated with miRNA-mediated inhibition of the PPARγ-NF-κB pathway through miR-27b [234].

The role of ginger in cancer prevention has been investigated in several randomized controlled trials. One of these studies involved patients at increased risk for colorectal cancer (CRC) [246]. The results suggest that daily consumption of ginger reduces proliferation in the crypts of normally appearing colorectal epithelium and promotes apoptosis. There are also numerous in vivo animal studies which have shown that ginger consumption is associated with a reduced risk of cholangiocarcinoma [247], liver [248], pancreatic [249] and gastric [250] cancer. Due to the poor water solubility of 6-gingerol, scientists have investigated the combination of 6-gingerol with nanoparticles which provided a prolonged drug effect [251] and, moreover, caused a significant regression of colon cancer when ginger was administered with calcium alginate [252]. Therefore, the use of 6-gingerol in chemoprevention holds a promising future with highly beneficial potential for the control and treatment of cancer [241].

### 6.4. Honokiol

Magnolia-derived agents have been strongly appreciated, even a hundred years ago, and medicaments being produced from cortex, cones and leaves of this plant were commonly used in ancient western medicine. One of the most common polyphenolic and low-toxic anticancer compounds isolated from *Magnolia officinalis* is honokiol (3′,5-di-(2-propenyl)-1,1′-biphenyl-2,4′-diol) [253].

According to recent in vitro studies, honokiol shows anticancer activity against miscellaneous cancer cell lines, e.g., Hep-G2 cells (hepatocellular carcinoma) [254], melanoma cell lines SK-MEL-2 [255], MeWo [256], pancreatic cancer [257] and human epidermoid A431 squamous skin cancer [258]. Honokiol inhibits NF-κB activation through the suppression of Akt and activation of IKK (inhibitor kinase) [253]. Furthermore, this agent plays a major role in inhibition of EGFR signaling in head and neck squamous cell carcinoma cells [259] and inhibition of mammalian target of rapamycin (mTOR) kinase responsible for controlling cell metabolism, growth and proliferation [253]. Moreover, due to the ability to decrease P-glycoprotein expression, honokiol is an object of interest for scientists involved in oncology as some studies have revealed that pretreatment with honokiol overcomes the resistance to cytostatics [260]. Wang X. et al. (2011) showed that honokiol may cross the blood-brain barrier and inhibit growth of human U251 xenograft glioma model [261]. Furthermore, this substance exhibits neuroprotective properties through a wide range of mechanisms and, therefore, is likely to become an interesting alternative for standard treatment for patients diagnosed with brain tumors. However, despite the aforementioned excellent properties, honokiol is not a perfect agent. It also has its disadvantages, namely poor bioavailability [262]. One of the last successful strategies to overcome this phenomenon was the use of nanomicellar particles which increase its bioavailability and anticancer effects [263].

### 6.5. Flavonoids

Flavonoids are a very diverse group of compounds displaying anti-proliferative effects in many cancer cell lines [264,265,266]. Thanks to their ability to neutralize the effects of oxidative stress as well as inhibit kinase activity and glucose uptake [267], flavonoids have caught the attention of researchers in recent years. Some flavonoids are capable of binding to cellular receptors, and thus alter cell proliferation [268]. Additionally, flavonoids are widely known as compounds altering multidrug resistance (MDR) phenomenon in cancer cells. For example, naringenin enhances the anti-tumor effect of doxorubicin by selectively modulating drug efflux pathways and increases the level of doxorubicin concentration in the cells overexpressing MDR associated proteins, and may find its application as an adjuvant drug in the treatment of human tumors [269]. Due to suboptimal pharmacokinetics and low bioavailability in the cancer sites, the application of naringenin in cancer is limited, which creates the necessity of using alternative drug delivery systems. The nanoparticle system of naringenin (NARNPs) was tested on human cervical carcinoma (HeLa) cells. The results showed that using NARNPs produces dose-dependent cytotoxicity as well as apoptosis. Moreover, NAPNPs caused alterations in mitochondrial membrane potential, increased intracellular ROS level and reduced intracellular GSH (glutathione) level [270].

#### 6.5.1. Green Tea Flavonoids

**Figure 4:**
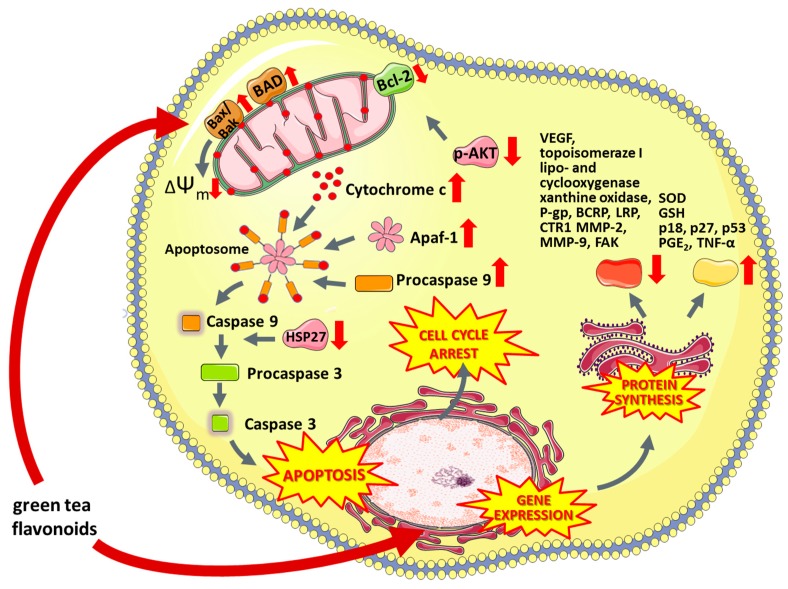
The multi-level anti-cancer activity of green tea flavonoids. ΔΨm—Mitochondrial membrane potential, Apaf-1—Apoptotic protease activating factor, BAD—Bcl-2-associated death promoter, Bak—Bcl-2-antagonist killer, Bax—bcl-2-like protein 4, Bcl-2—B-cell lymphoma 2, BCRP—ATP-binding cassette super-family G member 2, CTR1—High affinity copper uptake protein 1, FAK—focal adhesion kinase, GSH—Glutathione, HSP-29—heat shock protein 29, LRP—Low density lipoprotein receptor-related protein, MMP-2—Matrix Metalloproteinase 2 Protein, MMP-9—Matrix Metalloproteinase 2 Protein, p18—Cyclin-dependent kinase 4 inhibitor C, p27—Cyclin-dependent kinase inhibitor 1B, p53—tumor protein p53, p-AKT—Protein kinase B (phosphorylated), PGE2—Prostaglandin E2, P-gp—P-glycoprotein 1, SOD—Superoxide dismutase, TNF-α—tumor necrosis factor alpha, VEGF—Vascular endothelial growth factor.

Green tea contains many flavonoids such as epigallocatechin gallate (EGCG), epicatechin gallate, epigallocatechin, epicatechin and catechin [271]. They are among the strongest antioxidants and it has been proven that long-term consumption of green tea extracts increases the activity of glutathione S-transferase (GST) [272]. Drinking green tea increases the expression of oxidative stress enzymes, (e.g., superoxide dismutase) and inhibits the activity of lipo- and cyclooxygenase, xanthine oxidase, activator protein 1 and NF-κB transcription factors [273]. It is believed that the antitumor effect of green tea polyphenols is based on the induction of apoptosis [274], inactivation of transcription factors [275], cell cycle arrest in G1 phase [276] as well as inhibition of DNA synthesis and [277] activity of topoisomerase I [278] (Figure 4). Green tea compounds also affect formation of new capillaries, which limits the risk of tumor metastasis by reducing the supply of glucose and oxygen to cancer cells [279]. According to recent studies, polyphenols present in green tea affect multi-drug resistance by altering the expression of membrane proteins from the ABC family [274], breast cancer resistance protein (BCRP) [280,281], lung resistance protein (LRP) [281,282], ATP-dependent glutathione S-conjugate export pump [281,283], copper transporter CTR1 [284] or p-glycoprotein [281].

Numerous studies have identified EGCG to be epigenetically involved in modulation of carcinogenesis through DNA methylation (e.g., hypermethylation CpG islands) as well as histone modifications [285,286,287,288,289,290,291,292]. It is well known that EGCG possesses an inhibitory activity on DNMT [285,286,293], and HDAC [286,287,288,292] which reflects on reactivation of epigenetically silenced genes (p16 (INK4a), retinoic acid receptor beta (RARβ), O(6)-methylguanine methyltransferase (MGMT), human mutL homologue 1 (hMLH1) TIMP-3 [292] and acetylation glutathione-S transferase pi (GSTP1) [286,287]. An illustrative paper by Thakur et al. described proteasomal degradation of class I HDACs in human prostate cancer cells caused by green tea polyphenols treatment [287]. Notably, altered methylation patterns in promoter regions of tumor-suppressing genes caused re-expression of those in HeLa cells exposed to EGCG [294]. Another study demonstrated that EGCG decreased the invasive ability of cystic and adenoid cystic carcinoma SACC83 cells through the up-regulation of RECK protein [295]. Emerging evidence suggests that EGCG modulates polycomb group proteins (PcG) such as Bmi-1 and EZH2 [290,291] which corresponds to a decrease in repressive chromatin marks. A cutting-edge study by Chen et al. [296] presented comprehensive analysis of genome-wide methylation and mRNA expression in oral squamous carcinoma OSCC treated by EGCG. A total of 761 differentially methylated gene loci and 184 downregulated transcripts were reported and associated with the key metabolic pathways, as well as mitogen activated protein kinase (MAPK), Wnt signaling, and regulation of the cell cycle.

Several clinical trials have investigated the role of tea polyphenols in cancer prevention. Some studies suggest a protective role of green tea against lung [297], stomach [298], liver [299], colorectal [300], breast [301], and prostate [302] cancers. Drinking green tea has been shown to have beneficial effects in protecting the human body against oral premalignant lesions [303], oxidative DNA damage among smokers [304] as well as liver [305] and prostate cancers [306]. Today, there are also considered alternative methods for enhancing anti-tumor activities of green tea polyphenols, such as the use of special nanoparticles [307,308,309,310] and electrochemotherapy [311,312].

#### 6.5.2. Caffeic Acid Phenethyl Ester and Caffeic Acid

**Figure 5:**
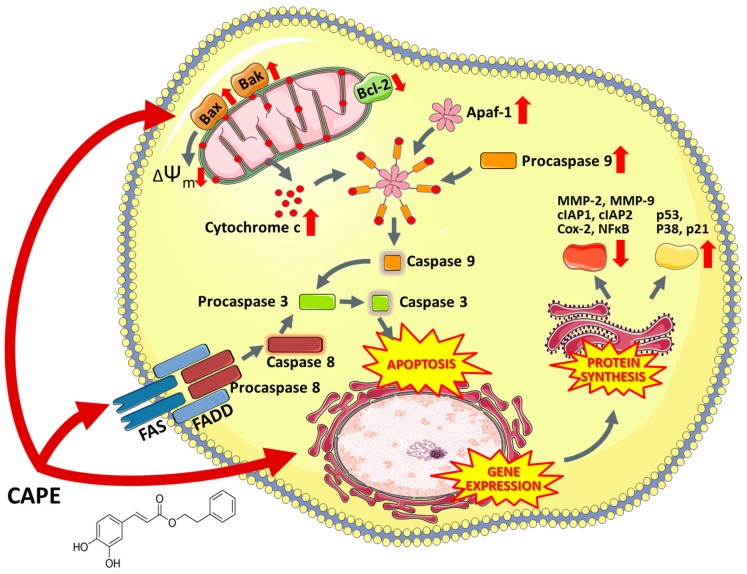
Mechanism of action of CAPE (Caffeic acid phenethyl ester) in cancer cells. ΔΨm—Mitochondrial membrane potential, Apaf-1—Apoptotic protease activating factor, Bak—Bcl-2-antagonist killer, Bax—bcl-2-like protein 4, Bcl-2—B-cell lymphoma 2, cIAP1—Cellular inhibitor of apoptosis protein-1, cIAP2—Cellular inhibitor of apoptosis protein-2, COX-2—Prostaglandin-endoperoxide synthase 2, CTR1—High affinity copper uptake protein 1, FAK—Focal adhesion kinase, FAS—Apoptosis antigen 1, FADD—Fas-associated protein with death domain, GSH—Glutathione, HSP-29—Heat shock protein 29, LRP—Low density lipoprotein receptor-related protein, MMP-2—Matrix Metalloproteinase 2 Protein, MMP-9—Matrix Metalloproteinase 2 Protein, NFκB—Nuclear factor kappa-light-chain-enhancer of activated B cells, p21—Cyclin-dependent kinase inhibitor 1, p38—p38 mitogen-activated protein kinases, p53—Tumor protein p53, p-AKT—Protein kinase B (phosphorylated).

Propolis is a compound of natural origin produced by bees—a mixture of bees’ saliva, beeswax and plant secretions [313]. Propolis consists of over 300 substances, most significant of which are flavonoids and phenolic acid. Among many active compounds found in propolis, caffeic acid phenethyl ester (CAPE) appears to be the most remarkable. Numerous studies have been performed to confirm its cytotoxic properties against tumor cells [314] as well as its antioxidant, anti-inflammatory, anticarcinogenic, antiviral, immunomodulatory, antihepatotoxic, neuroprotective, antiatherosclerotic activity [315,316,317,318,319,320,321,322]. The molecular targets of CAPE are proteins such as: ROS, COX-1, COX-2, NF-κβ, NFAT, AP-1, CYP2El, HIV1-integrase [315,321,323,324]. CAPE is also an apoptosis-inducing factor (Figure 5) which induces the apoptotic pathway via targeting p53, p38 and caspase-3 activity, inhibition of Bax and Bak and stimulation of Fas receptors [322,325,326,327]. Studies have reported CAPE to be a NF-κβ inhibitor; however, the induction of apoptosis by CAPE in PC-3 cells seemed to be entirely caspase-dependent [328]. In addition, CAPE may deplete intracellular supplies of GSH and trigger the ROS induced apoptotic pathway [329]. CAPE is also a selective inhibitor of both glutathione S-transferase [330] and matrix metalloproteinases (MMPs) [329,331]. In the HT1080 human fibrosarcoma cell line treated with CAPE, the compound exhibited a dose-dependent decrease in MMP activity [329]. What is more, CAPE has been proven to be able to affect oxidative stress pathways connected to p53-independent pathways to inhibit the growth of tumor cells.

Lee et al. (2006) were among the first to demonstrate that caffeic acid is involved in epigenetic mechanisms through the inhibition of DNMT in a non-competitive mechanism, resulting in decreased DNA methylation. Those findings were supported by in vitro experiments on human breast cancer cells (MCF-7 and MAD-MB-231) treated with caffeic acid, showing partial inhibition of the methylation of the promoter region of the RARb gene [332]. Another in vitro study on breast cancer reported the accumulation of acetylated histone proteins after incubation with CAPE, suggesting its HDAC inhibitory properties [333]. More recent evidence demonstrated a time-dependent reduction of global DNA methylated status induced by caffeic acid accompanied by a concomitant reduction of DNMT1 expression level [334].

## 7. Conclusions

Oncologists use a multidisciplinary approach to overcome cancers including surgical resection of tumors tissues, and radio- as well as chemotherapy. In many cases, surgery is the most substantial part of therapy and cytostatic agents or irradiation are often applied as an adjuvant or neoadjuvant treatment to increase effectiveness of the therapy and ensure better clinical prognosis. Nature has provided us with many substances displaying anticancer activity which have widely been applied in medicine, such as taxanes, vinca alkaloids, podophyllotoxin, camptothecin, anthracyclines, their derivatives and others [340]. In recent years, a vast number of natural compounds have been tested to establish their medical potential in cancer therapy [341]; however, only a small minority of these substances have qualified for clinical trials and treatment of cancer patients [26]. In recent years, polyphenols, (mostly green tea catechins) were investigated in patients suffering from prostate cancer; however, the collected data suggested that the action of the compounds was not directly aimed at cancer prevention [342]. On the other hand, green tea consumption does lower the risk of breast cancer [301]. Other clinical trials considering the described polyphenols included evaluation of ginger in colorectal cancer prevention. Additionally, some scientists studied the bioactivity of indole-3-carbinol from broccoli sprouts in preventing and treatment of breast, prostatic and pancreatic cancers [343,344]. The potential protective ability of phenetyl isothiocyanate against lung and prostate cancer or betulinic acid against cutaneous metastatic melanoma, has also been researched. Other clinical trials suggested that resveratrol might find an application in patients with colon cancers.

It is worth highlighting that more than half of cytostatics approved for medical treatment are nature-derived substances, their analogues and metabolites. Due to their non-selective activity and serious side effects, there is still a strong need to research and develop new medicines and substances of natural origin to improve human cancer treatment. The aforementioned agents present both low toxicity and potential selectivity against cancer cells. They are tolerable within the human organism even at high doses, in contrast to chemotherapeutics used in current cancer treatment. These substances may induce antimitotic activity via different mechanisms, particularly by disturbance of functionality of mitotic spindle or by inhibiting the activity of enzymes necessary in the DNA replication process. Some of these drugs may induce oxidative stress promoting cell death and limiting distant metastasis, affect activity of II phase enzymes, act as anti-angiogenic agents or inhibit cell migration. Additionally, some of those compounds may be used in photodynamic therapy or reversing of the multidrug resistance. Moreover, some studies suggest that the substances from medicinal plants prevent carcinogenesis [345], lower the risk of death and extend survival time among oncological patients [346]. Taking all this into account, and having in mind methods of increasing biodistribution of the agents (electroporation, sonoporation or encapsulation in special nanocarriers), they might become an interesting alternative to classical treatment. In some cases, synthetic modifications of these substances improve their anticancer activity, prolong their circulation time in the bloodstream or reduce toxicity [340]. Therefore, continued study using in vivo systems and improved clinical trials are necessary to establish the safety and clinical application of nature-derived medicines. Moreover, it is essential to use high-throughput drug screening technology such as NMR (nuclear magnetic resonance spectrometry), LC-MS (liquid chromatography-mass spectrometry), HPLC-MS (high-performance liquid chromatography-mass spectrometry) and others, to find new natural substances for forthcoming research [341]. Natural compounds remain an interesting tool in cancer treatment, thus we believe that more promising anticancer substances will be discovered which will allow for untapped scientific possibilities.

## Figures and Tables

**Table 1:** The mitotic inhibitors—a brief summary. Images of the chemical structures obtained from ChemSpider database [52]. ↑: upregulation/induction/stimulation, ↓: downregulation/inhibition.

Structure/Name	Mechanism(s)	Experimental Model	Compound Source	Ref.
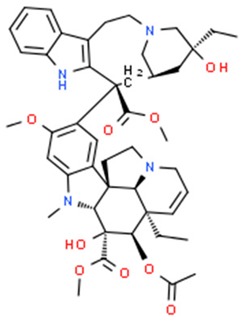 Vinblastine (above), vincristine, vindesine, vinorelbine	↓ of microtubule polymerization, ↑ apoptosis, ↑ microtubule depolymerization	HeLa cells, breast and lung cancer, Hodgkin’s disease, lymphosarcoma, chronic lymphocytic leukemia, acute lymphoblastic leukemia, Wilms’ tumor, rhabdo-myosarcoma, chorio-carcinoma, neuroblastoma	pink periwinkle plant *Catharanthus roseus*	[29,30,33,34,35,53,54,55]
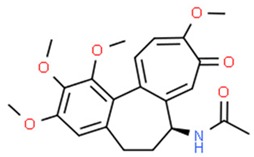 Colchicine	↓ mitosis, ↑ microtubules depolymerization	Due to the low therapeutic index, currently colchicine is not used as an anticancer agent	Meadow saffron *Colchicum autumnale* L.	[39,40,41,42,45]
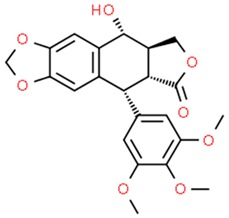 podophyllotoxin (above), etoposide and teniposide (semi-synthetic derivatives of podophyllotoxin)	↓ microtubule polymerization; ↓ topoisomerase II (etoposide and teniposide)	Etoposide and teniposide: germ-cell malignancies, lung cancer, Kaposi’s sarcoma, soft tissue sarcomas, leukemias, non-Hodgkin’s lymphoma, neuroblastoma	*Podophyllum peltatum* L., *Podophyllum emodi*	[50,56,57,58,59,60,61]
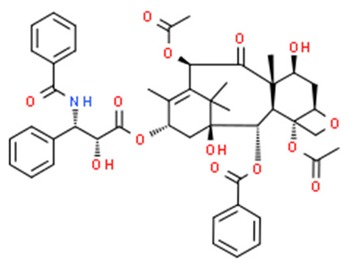 Paclitaxel (above), docetaxel	↑ microtubule polymerization	Kaposi’s sarcoma, breast, head, neck, lung, ovarian esophageal, prostate and bladder cancer	Pacific yew trees (*Taxus brevifolia*)	[62,63,64,65,66,67,68,69,70,71,72,73,74,75]

**Table 2:** Topoisomerases inhibitors—a brief summary. Images of the chemical structures obtained from ChemSpider database [52]. ↑: upregulation/induction/stimulation, ↓: downregulation/inhibition.

Structure/Name	Mechanism(s)	Experimental Model	Compound Source	Ref.
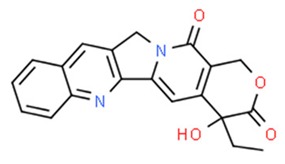 Camptothecin (above), topotecan and irinotecan (semisynthetic derivatives)	↓ topoisomerase I	Colorectal cancer, small cell lung cancer, leukemia	Tibetan tree *Camptotheca acuminate*	[79,80,81,83,84]
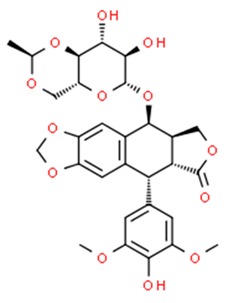 Etoposide	↓Topoisomerase II	Leukemia, small cell lung cancer, testicular tumors, Hodgkin’s lymphoma	*Podophyllum peltatum*	[85,86,87,88]

**Table 3:** Inducers of xenobiotics metabolism—a brief summary. Images of the chemical structures obtained from ChemSpider database [52]. ↑: upregulation/induction/stimulation, ↓: downregulation/inhibition DNMT—DNA methyltransferase, HDACs—histone deacetylase, MMPs—metaloproteinases, miRNAs—microRNA, DADS—diallyl sulfide.

Structure/Name	Mechanism(s)	Experimental Model	Compound Source	Ref.
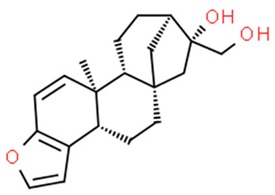 Kahweol (above) and other terpenes (diterpenes, kafestol)	↑ II phase enzymes (via NRF2 transcription factor)	Mice and rat liver and kidney cells	Green coffee seeds	[100,101,102,103,104]
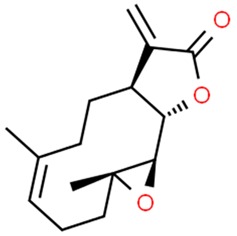 Parthenolide	↑ cell cycle arrest, cell differentiation, apoptosis, epigenetics: ↓ HDAC1 and DNMT1	Leukemia, cervical cancer, breast cancer	*Tanacetum parthenium* feverfew	[105,106,107,108,109,110,111]
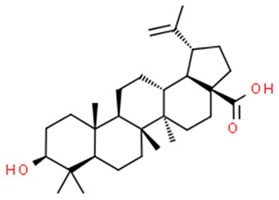 Betulinic acid	↑ apoptosis, mitochondrial membrane permeabilization, cell cycle arrest	Lung, cervical and ovarian cancer, melanoma, rhabdomyo-sarcoma, neuroblastoma, leukemia, epidermoid carcinoma	bark of *Betula pubescens*	[112,113,114,115,116,117]
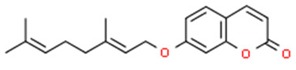 Auraptene (above) and other coumarins (furanocoumarin, pyranocoumarin)	↑ of II phase enzymes DHC: ↓HDAC (↓Rad52) ↓SIRT1 deacetylases	In vivo and in vitro animal model, yeast, human lymphoblastoid, colon, prostate ovarian, breast, non-small cell lung cancer, leukemia cell line	Citrus fruits and vegetables such as parsley, celery, parsnip	[118,119,120]
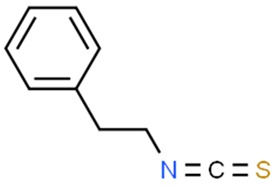 Phenethyl isothiocyanate (PEITC)	↑ of II phase enzymes, ↑DNMT1, ↑GSTP1; ↓HDAC, miRNAs modulation (cell proliferation, apoptosis and angiogenesis, cell invasiveness; ↓MMPs)	Animal and human cancer cells in vitro: breast, cervical, non-small cell lung. Prostate cancer, osteogenic sarcoma, myeloma cell lines	Spices, such as mustard, wasabi, and horseradish	[121,122,123,124,125,126,127,128,129,130,131,132,133,134,135,136]
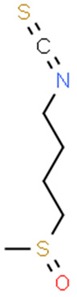 Sulforaphane	↑ II phase enzymes; ↑ apoptosis; ↓DNMT1s, ↓HDAC	Human cancer cells in vitro: breast, colon cancer, glioblastoma	Broccoli	[137,138,139,140,141]
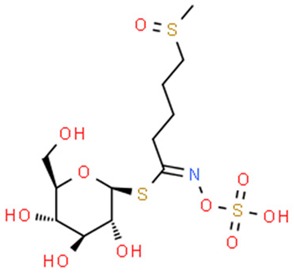 Glucoraphanin	↑ II phase enzymes	Weaker chemo-preventive properties than sulforaphane	Plant precursor of sulforaphane	[142,143,144]
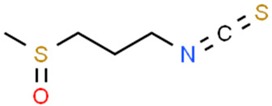 Iberin	↑ II phase enzymes; ↑apoptosis	Human cancer cells in vitro: breast, colon cancer, glioblastoma	Broccoli	[138,139,140,141]
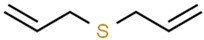 Allyl sulfide (above), diallyl sulfide,	↑ phase II enzymes DADS: ↓HDAC (↓ cell proliferation, ↑cell cycle arrest)	Rat intestine and liver cells, erythroleukemia, colon cancer cells	Genus *Allium*: garlic and onions etc.	[145,146,147,148,149,150,151,152,153]
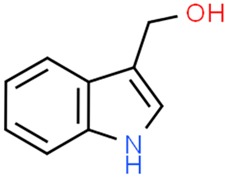 Indole-3-carbinol	↑ phase II enzymes ↓HDAC ↑ cell cycle arrest, apoptosis miRNAs modulation (↓cell invasiveness)	Rat liver cells, human cancer cells in vitro: breast, prostate, ovarian cancer	Vegetables of *Cruciferae* family, such as: cabbage, kale, brussels sprouts, broccoli	[154,155,156,157,158,159,160,161,162,163,164,165,166,167,168]

**Table 4:** Polyphenols—a brief summary. Images of the chemical structures obtained from ChemSpider database [52]. ↑: upregulation/induction/stimulation. ↓: downregulation/inhibition GBM—Glioblastoma multiforme, MDR—Multi-drug resistance, PcGs—Polycomb group proteins.

Structure/Name	Mechanism(s)	Experimental Model	Compound Source	Ref.
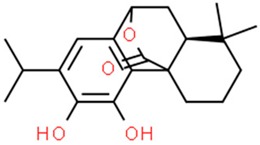 Carnosol	↓ CDKs, JAK2-STAT3, MAPK, Akt pathways; ↑ cycle arrest, p53 pathway	(GBM), breast, colon, skin, ovarian, prostate cancer, leukemia macrophage cell lines,	(*Rosmarinus officinalis),* oregano (*Origanum vulgare*), sage (*Salvia carnosa*	[201,202,203,204,205,206,207,208,210]
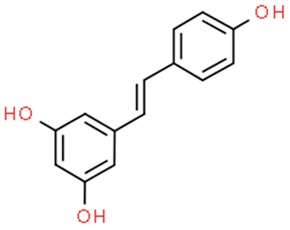 Resveratrol	Targeting COX (↓ tumor proliferation) ↑ cell cycle arrest ↓ NF-κB AP-1, HIF-1α, MMPs ↑ BRCA-1, NRF2, RASSF-1α; miRNAs modulation; ↓HDACs, DNMTs	many cancers in vitro and in vivo (e.g., prostate, breast, colorectal cancer) Clinical trials	*Polygonum cuspidatum* root, *Vitis vinifera* (common grape)	[22,216,217,220]
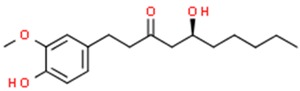 6-gingerol	↓angiogenesis, metastasis; ↑ apoptosis antitumor, antioxidant activity miRNAs modulation	Leukemia, breast, prostate, liver, colon, glioblastoma and pancreatic cancer	Rhizome of ginger (*Zingiber officinale* Roscoe)	[234,235,236,237,238,239,240]
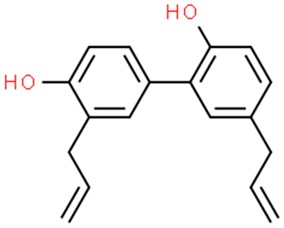 Honokiol	↑ apoptosis; ↓ EGFR signaling, P-glycoprotein	Hepatocellular carcinoma, melanoma, pancreas, epidermoid squamous skin cancer, glioma, head and neck squamous cancer	Cortex, cones and leaves of *Magnolia officinalis*	[254,255,256,257,258,261]
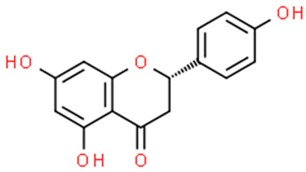 Naringenin	↓ proliferation, kinase and glucose uptake; ↑apoptosis; antioxidant activities; MDR modulation	Breast and colon cancer	Grapefruit, orange etc.,	[264,265,266,267,268,269,335,336]
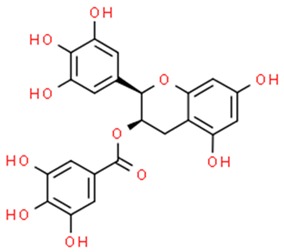 Green tea flavonoids epigallocatechin gallate (above), epicatechin gallate, epigallocatechin, epicatechin and catechin	↓ proliferation, angiogenesis, DNA synthesis, ↑apoptosis, cell cycle arrest; ↓HDACs, (↓cell invasiveness) DNMTs antioxidant activities; MDR modulation, PcGs modulation.	Many cancers in vitro and in vivo (e.g., lung ovarian, breast, prostate); Clinical trials	Green tea	[272,273,274,275,276,277,278,279,280,282,283,284,285,286,287,288,289,290,291,292,293,294,295,296,297,298,299,300,301,302,305,306]
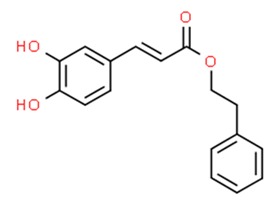 Caffeic acid phenethyl ester, caffeic acid	Cytostatic properties; ↑apoptosis; MDR modulation, ↓ MMPs; ↓HDACs, DNMTs	Laryngeal, pancreatic, brain, kidney, breast, lung, bladder, colorectal, prostate, head and neck cancer, melanoma	Propolis	[314,322,325,326,337,338,339]
